# Head-to-Head Comparison of Tissue Factor-Dependent Procoagulant Potential of Small and Large Extracellular Vesicles in Healthy Subjects and in Patients with SARS-CoV-2 Infection

**DOI:** 10.3390/biology12091233

**Published:** 2023-09-13

**Authors:** Marta Brambilla, Roberto Frigerio, Alessia Becchetti, Alessandro Gori, Marina Cretich, Maria Conti, Antonella Mazza, Martino Pengo, Marina Camera

**Affiliations:** 1 Centro Cardiologico Monzino IRCCS, 20138 Milan, Italy; marta.brambilla@ccfm.it (M.B.);; 2 National Research Council of Italy (SCITEC-CNR), 20133 Milan, Italy; 3 Istituto Auxologico Italiano IRCCS, 20149 Milan, Italy; 4 Department of Pharmaceutical Sciences, Università degli Studi di Milano, 20133 Milan, Italy

**Keywords:** small extracellular vesicles, large extracellular vesicles, tissue factor, factor Xa generation, COVID-19

## Abstract

**Simple Summary:**

Procoagulant extracellular vesicle (EV) concentrations have been found to increase in several diseases, but the relative contribution of small (sEVs) and large (lEVs) EVs to plasma prothrombotic potential is poorly defined. Our study shows for the first time that the concentration of tissue factor (TF)^pos^ sEVs is significantly higher than that of lEVs. Despite this, the TF-dependent procoagulant potential is primarily sustained by lEVs, although sEVs may also contribute to factor Xa generation when TF pathway inhibitor (TFPI) activity is reduced. Also, in thromboinflammatory conditions, such as COVID-19, the enhanced procoagulant potential that characterizes the infection is predominantly supported by lEVs, although both TFpos-sEVs and -lEVs increase during the acute phase of the disease and return to normal levels with infection remission. Therefore, circulating large EVs, instead of small ones, may be identified as a promising target for a future strategy aiming at reducing the procoagulant potential of blood.

**Abstract:**

The relative contribution of small (sEVs) and large extracellular vesicles (lEVs) to the total plasma procoagulant potential is not yet well defined. Thus, we compared total and TF^pos^-sEVs and -lEVs isolated from healthy subjects and COVID-19 patients during the acute phase of the infection and after symptom remission in terms of (1) vesicle enumeration using nanoparticle tracking assay, imaging flow cytometry, and TF immunofluorescence localization in a single-vesicle analysis using microarrays; (2) cellular origin; and (3) TF-dependent Xa generation capacity, as well as assessing the contribution of the TF inhibitor, TFPI. In healthy subjects, the plasma concentration of CD9/CD63/CD81^pos^ sEVs was 30 times greater than that of calcein^pos^ lEVs, and both were mainly released by platelets. Compared to lEVs, the levels of TF^pos^-sEVs were 2-fold higher. The TF-dependent Xa generation capacity of lEVs was three times greater than that of sEVs, with the latter being hindered by TFPI. Compared to HSs, the amounts of total and TF^pos^-sEVs and -lEVs were significantly greater in acute COVID-19 patients, which reverted to the physiological values at the 6-month follow-up. Interestingly, the FXa generation of lEVs only significantly increased during acute infection, with that of sEV being similar to that of HSs. Thus, in both healthy subjects and COVID-19 patients, the TF-dependent procoagulant potential is mostly sustained by large vesicles.

## 1. Introduction

Circulating extracellular vesicles (EVs) comprise a highly heterogeneous population of nano-sized particles released by almost all cell types, with a completely distinct biogenesis for small (sEVs) and large vesicles (lEVs) [1]. They play key roles in different physiological and pathological processes, being involved in coagulation, atherosclerosis onset and progression, angiogenesis, cell survival, and modulation of the immune response and inflammation [2,3,4]. The amount of circulating EVs increases in thrombotic, inflammatory or infectious diseases, and their molecular content changes with disease progression, reflecting the activation state of the involved cells and/or compartment [1]. Therefore, circulating EVs have emerged as both surrogate biomarkers of disease prognosis and potential pharmacological targets due to them being paracrine carriers able to influence target cells [5,6,7,8,9].

The thrombin generation capacity of EVs, particularly that of large ones, has been widely described and relies on the exposure of their membrane to both anionic phospholipids, which facilitate coagulation factors’ assembly and tissue factor (TF), the main activator of the blood coagulation cascade [10]. An increased number of TF^pos^-EVs, together with an enhanced procoagulant activity, has been reported in cardiovascular diseases, cancer and viral infections [5,11,12,13,14]. Notably, the relative contribution of sEVs and lEVs to the procoagulant potential of plasma is not yet well defined, with this assessment being quite challenging. Indeed, despite continuous technological improvements in the field, the characterization of sEVs is still particularly complex, mainly due to methodological issues in isolating and detecting pure sEV populations. In addition, preanalytical conditions, including the starting biological material (whether plasma or serum) and sample preparation, are often different among the different published studies [15,16,17,18,19,20]. This makes it difficult to compare data, so many efforts are being carried out to standardize procedures for the analysis of EVs.

Functional and/or antigen-based techniques have recently been applied to characterize sEV-associated TF in patients with SARS-CoV-2 infection [18,19,20,21]. The data, although sometimes contradictory, seem to identify TF^pos^-EVs as important contributors to the thrombotic events commonly observed in COVID-19 patients. These studies, however, lack a complete EV antigenic characterization, and, thus, they do not provide any clues on the cellular origin of EVs to identify the activated cell populations releasing them and, thus, to facilitate the development of interventions to modulate this event. Moreover, an in-depth analysis of the TF-dependent procoagulant potential of lEVs and sEVs—by using specific blocking antibodies against TF and its inhibitor, TFPI—would be worthwhile to provide insights into the contribution of these EV populations to the coagulation process in the effort to eventually target it.

Based on this rationale, in this study, we first compared total and TF^pos^-sEVs and lEVs isolated from healthy subjects in terms of (1) vesicle enumeration using nanoparticle tracking assay, imaging flow cytometry, and TF immunofluorescence localization in a single-vesicle analysis using microarrays; (2) cellular origin; and (3) overall procoagulant activity by analyzing the contribution of the physiological TF inhibitor, TFPI. These multidisciplinary integrated approaches were also applied to characterize TF^pos^-EVs in COVID-19 patients during the acute phase of infection and after symptom remission to unravel the contribution of sEVs and lEVs to the overall prothrombotic potential of the disease.

## 2. Materials and Methods

### 2.1. Patient Selection

This study took advantage from an existing biobank of plasma samples from COVID-19 patients enrolled during the acute phase of infection (*n* = 10; T0), whose characteristics are reported in Online Table I [14]. A cohort of patients who had recovered from SARS-CoV2 infection by 5 ± 2 months (*n* = 10; FU) and a group of healthy subjects (*n* = 10; HSs) were also enrolled. This study was approved by the Ethical Committee of the Institution (number 2020_06_16_18), and informed consent was obtained from all participants according to the principles of the Declaration of Helsinki. 

### 2.2. Blood Collection and Extracellular Vesicle (EV) Sample Preparation

Whole blood (WB) was drawn using a 19-gauge needle without venous stasis into citrate (1/10 volume of 0.129M sodium citrate) and processed within 15 min. Large (lEV) and small (sEV) extracellular vesicles were isolated via plasma differential centrifugation. For platelet-free plasma (PFP) for lEV analysis, WB was prepared according to the ISTH guidelines [22]. Briefly, WB was centrifuged twice at 2500× *g* for 15 min at room temperature (RT) to ensure complete platelet removal. PFP was collected into a fresh tube and stored at −80 °C until lEV analysis or diluted (1:3) in 0.1 µm pore-size-membrane-filtered PBS and centrifuged at 20,000× *g* for 20 min at RT to isolate lEVs for FXa generation analysis. For sEV isolation, ultracentrifugation (UC) was used: the supernatants derived from the PFP samples, previously centrifuged at 20,000× *g* for 20 min at RT, were ultracentrifuged at 100,000× *g* for 120 min at 4 °C to obtain sEV pellets [23].

### 2.3. Nanoparticle Tracking Assay (NTA)

The size distribution and concentration of sEV-containing samples were measured using NanoSight NTA (NS300) (Malvern Panalytical Ltd., Malvern, UK) equipped with a 488 nm laser. The samples were diluted (1:1000) in 0.1 µm pore-size-membrane-filtered PBS before analysis and analyzed at 25 °C following daily instrument calibration according to the manufacturer’s recommendation. Sample analysis was performed in triplicate over 60 s, with the camera level set to 14. Analysis was performed using the NTA v3.4 software (Malvern Panalytical Ltd., Malvern, UK).

### 2.4. Imaging Flow Cytometry

Multispectral imaging flow cytometric acquisition of EVs was performed using Amnis ImageStreamX MK II (ISx, EMD Millipore, Seattle, WA, USA) with fluidics set at low speed, sensitivity set to high, magnification at 60×, core size of 7 μm, the “Hide Beads” option unchecked prior to every acquisition to visualize speed beads in analysis, and all lasers set to maximum powers to ensure maximal sensitivity. sEV pellets were diluted in 0.10 µm pore-size-membrane-filtered PBS and, to avoid the risk of coincident particle detection, the EV samples were run at concentrations greater than 1010 objects/mL [24]. The sEV samples were labeled for 15 min at RT in the dark with saturating concentrations of centrifuged (17,000× *g*, 30 min, 4 °C) [25] α-CD81, α-CD9 and α-CD63, which are established sEV membrane markers. For lEV characterization, PFP was processed as previously described [5]. Briefly, fifty microliters of PFP was diluted in 0.22 µm pore-size-membrane-filtered PBS with D-phenylalanyl-L-prolyl-L-arginine chloromethyl ketone (PPACK; 15 µM) to prevent clot formation. To identify intact lEVs, the samples were incubated with calcein AM (10 µM) at 37 °C in the dark for 25 min. Saturating concentrations of centrifuged cell-specific population marker mAbs (α-CD41-PerCP Cy5.5 for platelets [5], α-CD14-PECy7 for monocytes [5], α-CD66-FITC for granulocytes [5], α-CD146-PE for endothelium [26]) and α-TF-BV421 and α-TFPI-CF647 MoAbs) were incubated for 15 min at RT in the dark. Fluorescence minus one (FMO) control was used to determine the cut-off between background fluorescence and positive populations.

FITC signals were collected in channel 2 (480–560 nm filter), PE signals in channel 3 (595–650 nm filter), and APC signals in channel 5 (660–745 nm filter). Upon each startup, the instrument calibration tool ASSIST^®^ was performed to optimize performance and consistency. All samples were acquired using the INSPIRE^®^ software (v4.0), and data analyses were performed using the ISx Data Exploration and Analysis Software (IDEAS^®^ v6.2). Technical controls included for all analysis of EVs comprised detergent lysis treatment performed by incubating the filtered PBS-diluted sEV samples in 0.1% Triton™ X-100 for 30 min at RT [27], the buffer controls without EVs and the unstained samples; moreover, to avoid false positive events, all antibodies used were run on ISx in filtered PBS alone to ensure antibody clumps were not present.

### 2.5. Single-Vesicle Microarrays 

Microarrays were performed using membrane-sensing peptide (MSP) immobilized on MCP-6-coated silicon chips, with 80 nm oxide layer thickness, using a non-contact S12 Spotter (Scienion Co., Berlin, Germany), depositing one drop for each spot according to [28].

The ExoView™ platform was used to analyze peptide microarray; label-free interferometric count, size measurement and fluorescence immunostaining were performed to detect single extracellular vesicles. The platform is able to provide quantitative and size information coupled to surface biomarker co-localization on each individual vesicle captured on the microarray spots. The simultaneous sizing of detected particles allowed us to focus on vesicles in a specific size range, namely 50–130 nm, and to confirm via fluorescence immunostaining the co-localization of TF with tetraspanins (CD9, CD81 and CD63) on such EV subpopulations.

A total of 50 µL of sEV diluted in PBS (1:100) was incubated for 2 h under static conditions on the array in a humid chamber. After incubation, the chips were moved onto a 24-well plate, and washes were performed two times for 2 min each at 300 rpm using an orbital shaker with filtered PBS. For fluorescence immune phenotyping, the chips were incubated with a mix of antibodies, including α-tetraspanin (CD9, CD63 and CD81) (Ancell, Bayport, MN, USA) and α-TF (HTF-1) labeled with CF555^®^ and CF647^®^ (biotium, San Francisco Bay Area, California, USA). Each antibody was diluted in filtered incubation buffer (Tris/HCl at 0.05 M at pH 7.6, NaCl at 0.15 M, Tween20 at 0.02%), and the chips were incubated dynamically at 300 rpm for 15 min. The chips were then washed with filtered PBS twice, followed by a rinse with MilliQ water, and dried.

The chips were then imaged using an ExoView R100 reader using the nScan 2.8.4 acquisition software. The data were then analyzed using the NanoViewer 2.8.4 ExoView Analyzer.

### 2.6. Platelet Isolation for In Vitro Experiments

Blood for in vitro experiments was collected via venipuncture from healthy subjects in tubes containing citrate (1/10 volume of 0.129 M sodium citrate). The specimens were centrifuged for 10 min at 100× *g* and platelet-rich plasma (PRP) was transferred to a new tube. For platelet isolation, PRP was diluted in Tyrode’s buffer (134 mM NaCl, 12 mM NaHCO_3_, 2.9 mM KCl, 0.34 mM Na_2_HPO_4_, 1 mM MgCl_2_, 10 mM HEPES, pH 7.4) 0.25% BSA (Sigma-Aldrich, St. Louis, MO, USA) and washed twice. Then, platelets were resuspended in Tyrode’s buffer with 0.25% BSA and 2.5 mM CaCl_2_. Spontaneously released large and small platelet-derived extracellular vesicles were prepared as reported above.

### 2.7. FXa Generation

The factor Xa generation assay (Actichrome^®^ TF) was performed according to the manufacturer’s protocol with minor modifications. Briefly, sEVs and lEVs, isolated as described above, were resuspended in 0.1 µm pore-size-membrane-filtered PBS, mixed with factor VIIa (1.75 µg/mL) and factor X (7 µg/mL), and incubated at 37 °C for 15 min. The specific contribution of TF was further evaluated by performing the assay in the presence of neutralizing α-TF (HTF-1; Invitrogen, Waltham, MA, USA; 8 µg/mL) and α-TFPI (ADG4903; ImmBioMed, Pfungstadt, Germany; 8 µg/mL) antibodies. Sample absorbance was read at 405 nm using a microplate reader (Infinite M Plex, Tecan, Männedorf, Switzerland) after the addition of a specific chromogenic substrate (Spectrozyme-FXa). The amount of active TF was calculated based on a standard curve generated using known amounts of human TF. TF-dependent FXa production was calculated as the difference between total FXa and TF-independent FXa, i.e., produced in the presence of αTF-neutralizing Abs.

### 2.8. Statistical Analysis

The results are expressed as mean ± standard deviation (SD) and were analyzed using Student’s paired *t*-test or Mann–Whitney U test, as appropriate. A *p*-value of 0.05 was considered statistically significant. Analyses were performed using the SPSS statistical package (v9.4).

## 3. Results

### 3.1. Plasma EV Concentration and Cellular Origin Evaluation

The plasma concentration of circulating sEVs was assessed using both nanoparticle tracking analysis (NTA), the most currently used method for the measurement of number and size of sEVs, and imaging flow cytometry, which provides a count of the analyzed events based on the tetraspanin antigenic profile. As reported in Table 1, the mean concentration of total sEVs in the HSs was 2.27 ± 0.13 × 10^8^/µL. 

**Figure 1:**
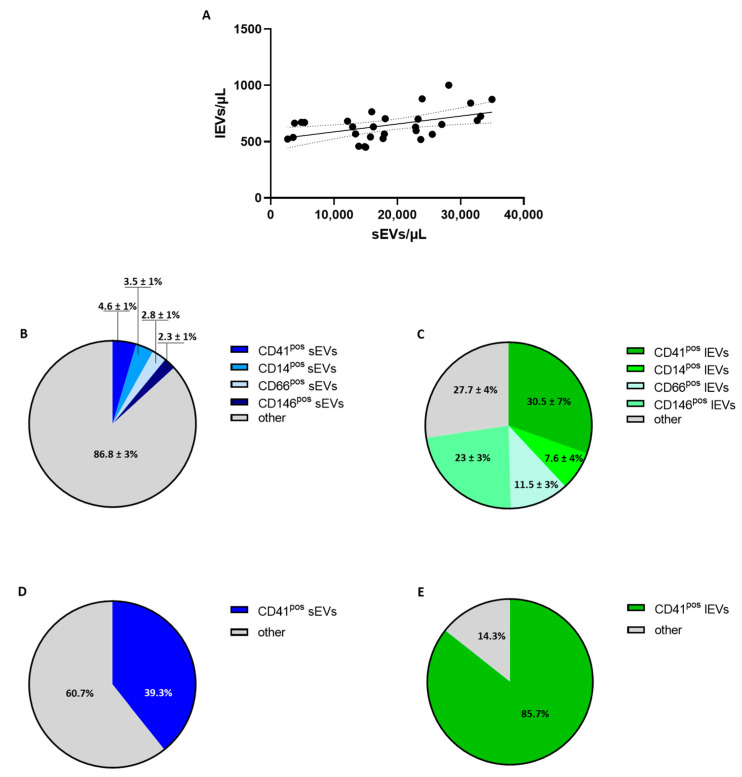
Analysis of sEV and lEV cellular origin. (**A**) Correlation between circulating concentration of small and large vesicles in healthy subjects. Platelet (CD41)-, monocyte (CD14)-, granulocyte (CD66)- and endothelium (CD146)-derived small (sEVs; **B**) and large (lEVs; **C**) extracellular vesicle distributions in healthy subjects (*n* = 10) were evaluated using flow cytometry. Percentages of CD41^pos^ small (sEVs; **D**) and large (lEVs; **E**) vesicles spontaneously released in vitro from isolated washed platelets. Individual data or percentage (%) ± SD are shown.

The flow cytometry analysis showed that among these vesicles, those expressing the tetraspanins CD81, CD63 and CD9, which are classical exosome markers, had a concentration of 1.98 ± 0.6 *×* 10^4^/µL, a concentration which was about 4 orders of magnitude lower than that obtained using NTA and 30 times greater than the amount of calcein^pos^ lEVs (Table 1). Interestingly, the total number of CD81/CD63/CD9^pos^ sEVs positively correlated with that of calcein^pos^ lEVs (r = 0.4840; *p* = 0.0078; Figure 1A).

Among the potential cells releasing EVs tested, the data show that platelets, monocytes, granulocytes and endothelial cells released both sEVs and lEVs, with those derived from platelets being the most abundant (Table 2).

Of note, the expression of these cell population markers was a feature limited to only 13% of sEVs, while their expression was significantly more common for calcein^pos^ lEVs (~70%) (Figure 1B,C). 

To confirm that the paucity of population antigens is a hallmark of sEVs, we estimated the percentage of sEVs carrying CD41, a specific platelet-population marker, that were spontaneously released in vitro from isolated washed platelets. The data show that less than 40% of sEVs expressed CD41, while almost all lEVs were CD41^pos^ (Figure 1D,E). Of note, a similar feature was observed when EVs released from platelets stimulated with ADP (36.3% ± 3.3% for CD41^pos^ sEVs and 84.2% ± 4.2% for CD41^pos^ lEVs) or arachidonic acid (35.9% ± 1.6% for CD41^pos^ sEVs and 85.5% ± 4.2% for CD41^pos^ lEVs) were characterized. 

### 3.2. Plasma Concentration of TF^pso^ -sEVs and -lEVs and Procoagulant Activity Assessment

**Figure 2:**
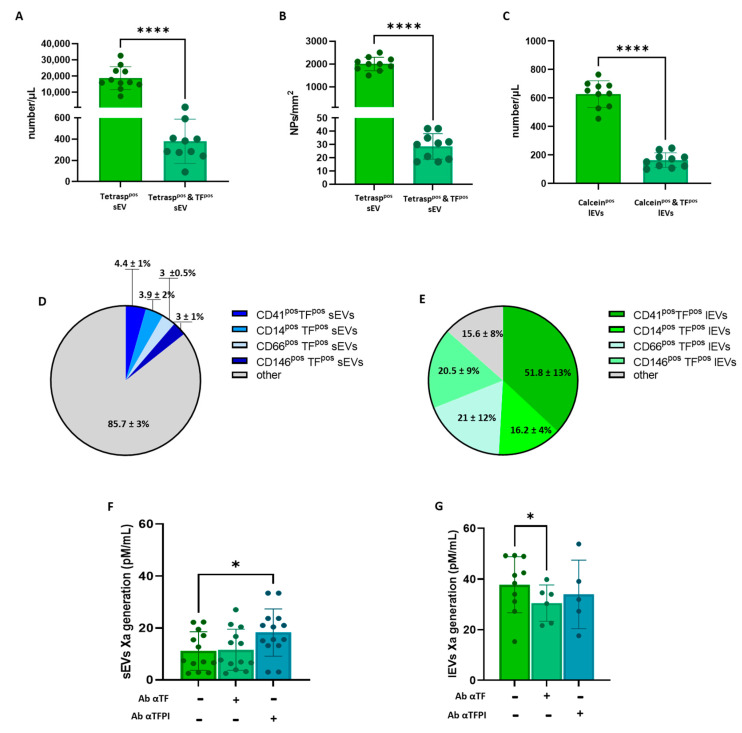
Assessment of circulating tissue factor-positive plasma extracellular vesicles and analysis of their procoagulant potential. Concentration of total (Tetraspan^pos^) and tissue factor-positive (TF^pos^) small extracellular vesicles (sEV) from healthy subjects (*n* = 10) analyzed using (**A**) flow cytometry and (**B**) multiparametric peptide microarray. (**C**) Concentration of total (Calcein^pos^) and tissue factor-positive (TF^pos^) large extracellular vesicles (lEV) from healthy subjects (*n* = 10) assessed using flow cytometry. Percentages of tissue factor-positive (TF^pos^), small (**D**) and large EV (**E**)-expressing platelet (CD41), monocyte (CD14), granulocyte (CD66) and endothelium (CD146) population markers. Factor Xa generation capacity of small (**F**) and large EVs (**G**) of healthy subjects (*n* = 5–10) measured in the absence or presence of neutralizing αTF or αTFPI antibody. Individual data are shown. Mean number ± SD, mean number of particles per mm^2^ (NP) ± SD, or percentage (%) ± SD are reported. Results were analyzed using Student’s paired *t*-test or Mann–Whitney U test, as appropriate. * *p* < 0.05; **** *p* < 0.0001.

The number of TF^pos^-sEVs evaluated via flow cytometry was 380 ± 208/μL and accounted for less than 2% of tetraspanin^pos^ sEVs (Figure 2A). A similar ratio was confirmed when sEVs were counted using the peptide microarray technology (Figure 2B). Unlike sEVs, the percentage of TF^pos^-lEVs accounted for 25% of total calcein^pos^-lEVs (Figure 2C), with their absolute amount being lower than that of sEVS (165 ± 52/μL vs. 380 ± 208/μL; *p* = 0.0053). Overall, while the concentration of total sEVs exceeded that of lEVs by ~30 times, the concentration of TF^pos^-sEVs only doubled that of lEVs, and they did not correlate (r = 0.361; *p* = 0.305). 

As far as their cellular origin is concerned, similar to what was observed for total EVs, less than 4% of TF^pos^-sEVs carried the population markers (Figure 2D). Cell-specific antigens were, by contrast, expressed by almost all (more than 90%) lEVs, with those derived from platelets showing the largest amount (43%; Figure 2E). 

To gain insight into the EV-associated procoagulant activity, factor Xa (FXa) generation was measured. The results showed that lEVs isolated from 1 mL of plasma had more than three-fold greater procoagulant potential than sEVs contained in the same plasma volume (36.76 ± 11.09 and 11.16 ± 7.42 pM/mL, respectively; Figure 2F,G). 

The TF-dependent FXa generation was, thus, assessed using a specific antibody that neutralizes the activity of TF and TFPI, the TF physiological inhibitor. FXa generated by lEVs only, and not by sEVs, was significantly inhibited (−30%) by the neutralizing αTF antibody (Figure 2F,G). Interestingly, however, a 63% increase in the FXa generation capacity of sEVs was measured upon the inhibition of TFPI activity; the αTFPI antibody, by contrast, did not affect the amount of FXa generated by the larger vesicle population.

These findings, therefore, suggest that TF carried by both lEVs and sEVs is functionally active, although the procoagulant potential of TF^pos^-sEVs is hindered by TFPI activity. 

### 3.3. Procoagulant Potential of sEVs and lEVs in Patients with SARS-CoV2 Infection

Recently, it has been shown that total and TF^pos^ -small and -large EVs are increased during the acute phase of SARS-CoV2 infection, thus potentially contributing to the procoagulant phenotype of the disease [14,29]. No data on their relative contribution to this process are available so far. Thus, we examined the relative capacity of small and large EVs to generate thrombin in COVID-19 patients.

**Figure 3:**
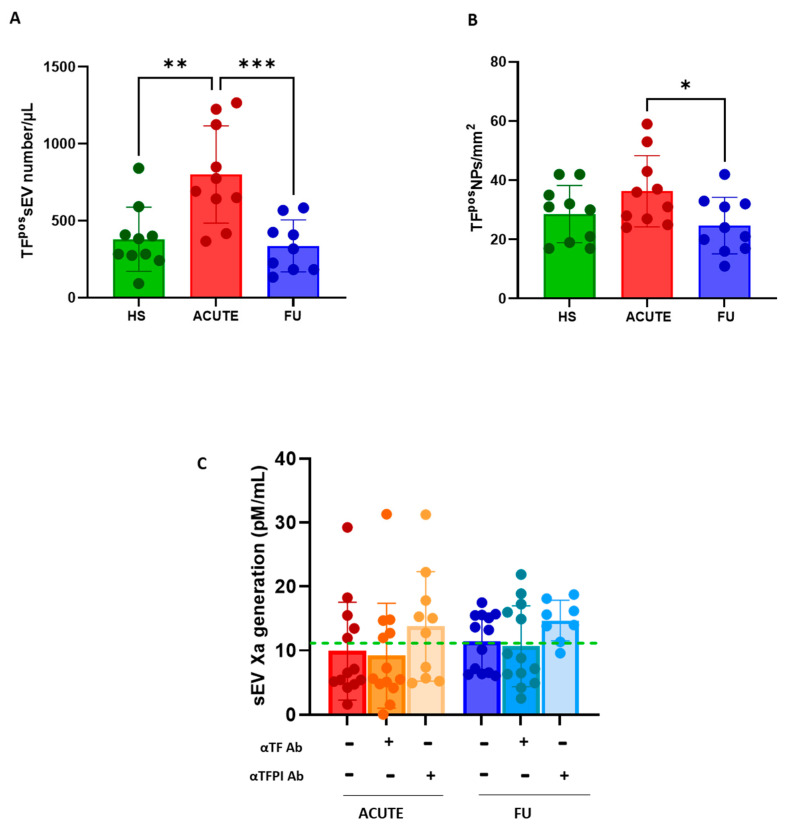
Procoagulant potential of small EVs in patients with SARS-CoV2 infection. Levels of tissue factor-positive small EVs (TF^pos^-sEVs) was analyzed using (**A**) flow cytometry and (**B**) multiparametric peptide microarray in patients with SARS-CoV2 infection enrolled during the acute phase (ACUTE; *n* = 10; red bars) and at 6-month follow-up (FU; *n* = 10; blue bars). Healthy subjects (HSs; *n* = 10; green bars) were analyzed for comparison. (**C**) Factor Xa generation capacity of sEVs was measured in the absence or presence of neutralizing αTF or αTFPI antibody. The dot line indicates the mean value measured in the HSs as a reference. Individual data and mean or mean number of particles per mm^2^ (NP) ± SD are reported. Results were analyzed using Student’s paired *t*-test or Mann–Whitney U test, as appropriate. * *p* < 0.05; ** *p* < 0.01; *** *p* < 0.001.

Compared to the HSs, patients during the acute phase of infection had a significantly greater number of TF^pos^-sEVs, as assessed using flow cytometry (Figure 3A) and the peptide microarray technology (Figure 3B). The levels of TF^pos^-sEVs reverted to physiological concentrations upon the remission of symptoms (Figure 3A,B).

Of note, the increased overall number of TF^pos^-sEVs during the acute phase of the disease was not paralleled by an increase, compared to the HSs, in FXa generation capacity, which remained unchanged even during disease remission (Figure 3C). Interestingly, in contrast to what was observed in the HSs, the presence of the neutralizing αTF antibody reduced Xa generation (−2.6 ± 2 pM/mL; *p* = 0.047) in 3 out of 10 acute COVID-19 patients, thus highlighting a functional TF activity.

Moreover, in the acute COVID-19 patients, the effect of the neutralization of TFPI activity accounted for an increase of 3.9 ± 2.8 pM/mL in FXa production (+39%), which was lower compared to that measured in the HSs (7.1 ± 2.2 pM/mL FXa, +63%). Since the effect of TFPI antibody on procoagulant activity is directly related to the amount of protein, these data indirectly point to a lower TFPI expression in COVID patients compared to HSs.

**Figure 4:**
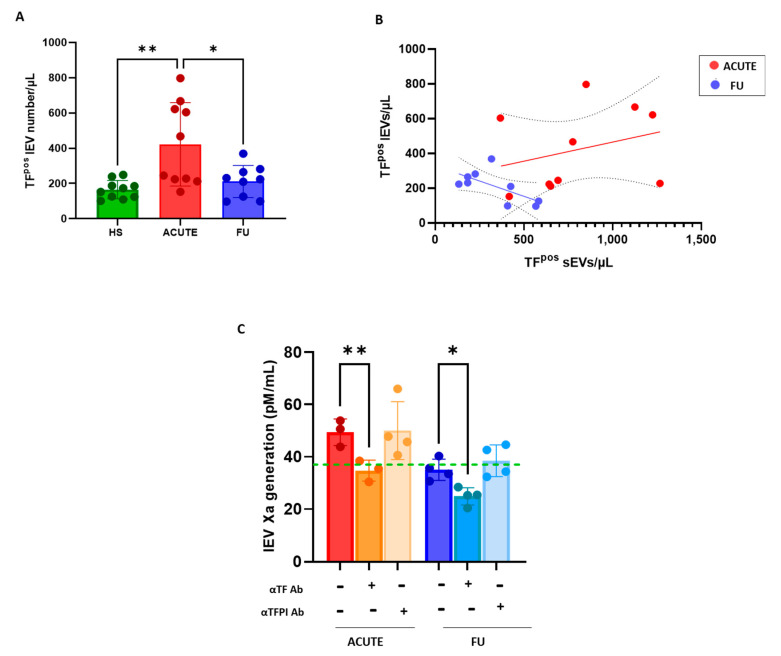
Procoagulant potential of large EVs in COVID-19 patients during acute infection and at 6-month follow-up. Levels of tissue factor-positive large EVs (TF^pos^-lEVs) was analyzed using (**A**) flow cytometry in patients with SARS-CoV2 infection who were enrolled during the acute phase (ACUTE; *n* = 10; red bars) and at a 6-month follow-up (FU; *n* = 10; blue bars). Healthy subjects (HSs; *n* = 10; green bars) were analyzed for comparison. (**B**) Association between TF^pos^-sEVs and -lEVs in acute and FU COVID-19 patients (**C**); factor Xa generation capacity of lEVs was measured in the absence or presence of neutralizing αTF or αTFPI antibody. The dot line indicates the mean value measured in HSs as a reference. Individual data and mean ± SD are reported. Results were analyzed using Student’s paired *t*-test or Mann–Whitney U test, as appropriate. * *p* < 0.05; ** *p* < 0.01.

The acute COVID-19 patients had a 2-fold greater concentration of TF^pos^-lEVs than the HSs (Figure 4A). The amount of TF^pos^-lEVs significantly correlated with that of TF^pos^-sEVs (r = 0.5181; *p* = 0.0231; Figure 4B), unlike what was previously observed in the HSs. As for sEVs, the levels TF^pos^-lEVs returned to concentrations similar to the HSs at the 6-month follow-up (Figure 4A), losing the association with sEV concentration (r = −0.6533; *p* = 0.0564; Figure 4B).

Interestingly, unlike what was observed for the sEV population, in the acute COVID-19 patients, the increased lEV levels were paralleled by an increase in TF-dependent FXa generation when compared to the HSs (αTF-neutralizing antibody effect: 14.7 ± 1.1 pM/mL and 4.2 ± 0.7 pM/mL in acute COVID patients and HSs, respectively; *p* = 0.001). Six months after recovery, the procoagulant potential was significantly reduced (−30%) compared to the acute phase, reaching the levels measured in the HSs (34.8 ± 4.9 and 36.7 ± 11.1 pM/mL, respectively; Figure 4C). As evidenced in the healthy conditions, inhibition of TFPI activity did not result in significant changes in the concentration of FXa produced by lEVs, both during the acute phase and at the FU. Despite this, the overall TF-dependent FXa generation supported by lEVs significantly exceeded that of sEVs at both the acute phase (lEV: 14.7 ± 1.1 pM/mL vs. sEV: 3.9 ± 2.8 pM/mL; *p* = 0.022) and FU (lEV: 10.2 ± 4 pM/mL vs. sEV: 3.2 ± 2.3 pM/mL; *p* = 0.05).

Finally, the expressions of platelet and leucocyte population markers in both TF^pos^-sEVs and -lEVs were analyzed using flow cytometry. As shown in Table 3, greater levels of both TF^pos^-sEVs and lEVs derived from platelets were highlighted during the acute infection phase compared to the HSs. A similar trend was also observed in monocyte- and granulocyte-derived EVs. Upon the remission of symptoms, the levels of all of these EVs reverted to the concentrations found in the HSs (Table 3).

**Figure 5:**
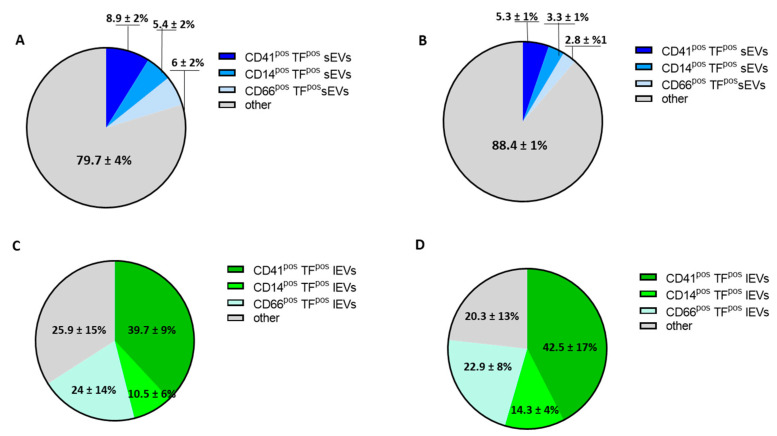
Evaluation of cellular origin of tissue factor-positive sEVs and lEVs in COVID-19 patients during acute infection and at 6-month follow-up. Percentages of tissue factor-positive (TF^pos^) (**A**,**B**) small and (**C**,**D**) large EV-expressing platelet (CD41), monocyte (CD14) and granulocyte (CD66) population markers during acute SARS-CoV-2 infection (**A**,**B**) and at 6-month follow-up (**B**,**D**). Data are reported as percentage ± SD.

Of note, despite the differences in the measured EV concentrations, the relative percentages of both sEVs and lEVs derived from platelets, monocytes and granulocytes were comparable both during the acute phase of the disease (Figure 5A,C) and at 6-month FU (Figure 5B,D) when compared to the HSs. 

Based on these findings, it can be speculated that the cell activation status that characterizes the acute phase of the disease equally induces all considered cellular compartments to release EVs. Thus, despite an overall increase in the number of EVs, the relative contribution of each cell population remains almost unchanged.

## 4. Discussion

In the current study, we characterized for the first time the physiological signature, in terms of concentration and cellular origin, of circulating extracellular vesicles, taking into account both the patterns of large and small vesicles and defining which of these contributed most to the plasma TF-dependent procoagulant potential. The data indicate that in healthy subjects, (1) the number of sEVs is 30 times greater than that of lEVs; (2) both large and small EVs are mainly derived from platelets among the possible cellular origins analyzed; and (3) the TF-dependent procoagulant potential is mostly carried by large vesicles, although sEVs could also contribute to FXa generation when TFPI activity is blunted.

Finally, to test whether pathological conditions could modify this pattern, the procoagulant contribution of two classes of EVs was evaluated in the context of SARS-CoV-2 infection. The data reported in this study show that the increased procoagulant potential that characterizes COVID-19 is supported mainly by large EVs, similar to what was observed in HSs, although both the increases in small and large TF^pos^ vesicles during the acute phase of this disease revert to physiological levels with infection remission.

Since the discovery of EVs, extensive research has been performed in this field, mainly on the characterization of the origin and functions of large size vesicles [30]. The identification of their protein and lipid composition has highlighted their importance in disease progression and as a diagnostic tool [31], as it may provide information on cell types that release EVs and if cells undergo pathophysiological changes such as activation, differentiation and replication. Unlike lEVs, in-depth characterization of sEVs is more challenging. sEVs were first characterized in the early 1980s, approximately 10 years after the initial studies on lEVs [32,33,34]. Although numerous studies have investigated the functions of sEVs, the definition of their cellular origin is still limited [35]. This is also partly due to technical issues related to the sensitivity of the instruments available to date. Indeed, the analysis of sEVs is often hampered by technical challenges in isolating, detecting and sizing these smaller vesicles and by the complex composition of blood, resulting in low exosomal purity due to the concomitant co-isolation of lipoproteins and protein aggregates of a similar size or density as EVs [3,15,36]. In this study, these limitations were circumvented through the use of high-sensitive cytofluorimetric techniques, such as imaging flow cytometry. Indeed, due to the use of a charge-coupled device camera that reduces background noise, imaging flow cytometry allows accurate detection and characterization of sEVs [24,36,37]. Thus, compared to the available published data, the added value of this paper is that, for the first time, large and small vesicles from the same plasma samples were characterized and then compared using the same technology, thus providing information on the subpopulations and molecular heterogeneity of EVs at a single-vesicle level. 

Nevertheless, as the MISEV 2018 guidelines for EV characterization suggest that sEVs have to be analyzed using different but complementary techniques [38], flow cytometry data were corroborated with both NTA and more specific microfluidic lab-on-a-chip platforms for sEV high-throughput analysis to phenotype sEVs. NTA is among the most widely used technologies for sEV count and size [39]. However, it should be emphasized that NTA, at least in the scatter mode, is not able to discriminate EVs from other light-scattering entities, such as lipoproteins or protein aggregates, and thus may not be sufficiently sensitive. Indeed, using this tool, we obtained a count of vesicles approximately four orders of magnitude greater than that identified using flow cytometry.

We achieved higher sensitivity through the use of microarrays based on the Single Particle Interferometric Imaging Sensor (SP-IRIS) technique coupled to the ExoView instrument for fluorescence detection. With this approach, we used membrane-sensing peptide (MSP) ligands as molecular baits for sEVs spotted on the microarray chips; MSP has an affinity for specific membrane traits of small EVs (50–130 nm) such as charge, lipid defects, curvature [28], and small lipid nanovesicles [40]. By using the ExoView platform, we evaluated the microarray-captured sEVs’ surface expression of TF while simultaneously confirming their EV nature by detecting the tetraspanins CD9, CD63 and CD81. The results obtained using this technology perfectly matched the flow cytometry results, thus validating the accuracy of sEV analysis via flow cytometry and its possible use for in-depth antigenic signature characterization.

Several studies have suggested that EVs are suitable as biomarkers due to their biological significance and easy accessibility from a broad range of body fluids [5,6,7,8]. Despite this, it is challenging to demonstrate the cellular origin of EVs. In this study, we characterized the cellular origin of both sEVs and lEVs. However, while lEVs were almost all positive for the antigens tested, only 15% of sEVs carried the cell population markers examined. This might be due to the different biogenesis of the two types of vesicles. Unlike lEVs, which are secreted via direct budding of the plasma membrane, sEVs are generated through intraluminal invagination of early endosomes, giving rise to multivesicular bodies that are released into the extracellular environment upon the fusion of these multivesicular bodies with the plasma membrane [41]. Thus, it is conceivable that, unlike lEVs, they do not incorporate surface population markers during their formation. This would explain the limited antigenic characterization of sEVs.

Once they are released, EVs participate in multiple pathophysiological processes [3,42], including the thrombotic process through, in both sEVs and lEVs, the presence of TF and phosphatidylserine, the two key players in the activation and amplification of the coagulation cascade, respectively [20]. In this study, we showed that, despite the higher plasma concentration of TF^pos^-sEV compared to lEVs, the TF-dependent procoagulant potential, as measured through the use of neutralizing antibodies to distinguish between TF-dependent and TF-independent activities, was predominantly carried by lEVs. Of note, however, unlike in the lEV population, the neutralization of TFPI activity in sEVs accounted for an increase in FXa production, suggesting that sEVs could also carry procoagulant TF, whose activity is hindered by TFPI. Whether the reduced procoagulant potential of sEVs may be due to the presence of a non-functionally active TF or to the presence of a high number of TFPI^pos^ vesicles able to inhibit TF activity, as shown in this study, needs to be further investigated.

The predominant TF-dependent activity of large EVs was also observed when the contribution to procoagulant activity was measured in the context of thromboinflammatory diseases, such as SARS-CoV-2 infection. We previously provided evidence that COVID-19 is characterized by a major alteration in the hemostatic balance toward a procoagulant phenotype, which is also supported by increased levels of TF^pos^ platelets and lEVs [14]. The data reported in this study further extend our knowledge in this clinical setting by showing that lEVs only carry TF-dependent FXa generation capacity, which was found to be significantly greater compared to that of HSs and recovered patients, despite the fact that both TF^pos^-lEVs and -sEVs doubled during the infection.

These data are consistent with those provided by Krishnamachary et al., who similarly demonstrated a positive correlation between the TF activity associated with lEVs and the severity of illness and length of hospitalization during the acute phase of infection [19]. Interestingly, the longitudinal analysis of the patients enrolled in our study showed that resolution of the disease led to the restoration of physiological concentrations and function of procoagulant lEVs. These data highlight that TF^pos^-lEVs, as opposed to sEVs, could significantly contribute to the thrombotic events that characterize this disease.

It is worth mentioning that, when the activity of the TF inhibitor, TFPI, is blunted by a neutralizing antibody, the FXa capacity of sEV increases, although not reaching the lEV values and to a lesser extent than in HSs. It can be speculated that a lower concentration of TFPI^pos^ sEVs, which is able to limit the procoagulant potential associated with sEVs, is present in COVID-19 patients when compared to HSs.

An additional important finding of this study is the evidence that platelets appear to be the primary source of circulating EVs, particularly of procoagulant EVs, among those analyzed in this study. It is widely recognized over the years that functionally active TF is present in a subpopulation of platelets, which are, therefore, able to promote and maintain the coagulation process [43]. The number of prothrombotic TF^pos^ platelets in different thrombophilic conditions, such as CVD, tumors, and infections, has been extensively documented [5,14,44]. In these pathological conditions, the levels of platelet-derived TF^pos^-EVs, mainly lEVs, are also found to be increased. Suades and collaborators showed that the levels of platelet-derived EVs enhance thrombosis on atherosclerotic plaques [45] and can predict adverse cardiovascular outcomes [6]. This makes such EVs not only important diagnostic markers but also early indicators of a particularly activated cell population, thus deserving targeted drug therapy. In this regard, there is evidence of the benefit of antiplatelet drug treatment in regulating procoagulant phenotype, even in SARS-CoV-2 infection [14,46].

## 5. Study Limitations

The results of this study should be evaluated in the context of their limitations. First, the antigenic characterization of EVs did not include an analysis of those originating from erythrocytes. In physiological conditions, red blood cell (RBC)-derived EVs account for 4–8% of all circulating EVs [47]. However, in pathological conditions, the concentration of RBC-derived EVs in circulation can increase, particularly in the presence of an alteration of the redox balance. However, as red blood cells lack an endosomal network, they are able to release only lEVs via plasma membrane budding [48]. Second, less than 20% of circulating sEVs have been characterized in terms of their cellular origin. It is likely that an assessment of their protein content would lead to a more complete understanding of both their source and their effects on target cells. Indeed, sEV molecular cargo is cell specific, which is regulated by tissue physiology and cellular function and is essential to their bioactivity [49]. Finally, an enumeration of TFPI^pos^ EVs, as well as of those derived from endothelium in COVID-19, was not performed in this study since they were not included among the variables to be assessed at the time of protocol approval by the Ethics Committee. Their analysis could have been useful in complementing and supporting the functional data in defining the procoagulant contribution of EVs in COVID-19. 

## 6. Conclusions

In conclusion, this study shows for the first time that the plasma concentration of TF^pos^ -sEVs far exceeds that of lEVs. Despite this, the TF-dependent procoagulant potential is mainly sustained by lEVs, although sEVs could also contribute to FXa generation in conditions where TFPI activity is reduced. 

Thus, circulating lEVs, rather than small ones, can be identified as a possible promising target for a future strategy aiming at reducing the procoagulant potential of blood.

## Figures and Tables

**Table 1:** sEV and lEV plasma concentration.

	Methodology	No./µL
Total sEVs	NTA	2.27 ± 0.13 × 10^8^
CD81/63/9^pos^ sEVs	flow cytometry	1.98 ± 0.6 × 10^4^
Calcein AM^pos^ lEVs	flow cytometry	0.63 ± 0.09 × 10^3^

NTA: nanoparticle tracking analysis; sEVs: small extracellular vesicles; lEVs: large extracellular vesicles; No.: number. Data are reported as mean ± SD.

**Table 2:** Circulating EVs’ cellular origin.

	sEVs (No./μL)	lEVs (No./μL)
Platelet-derived	922 ± 198	192 ± 44
Monocyte-derived	703 ± 285	48 ± 20
Granulocyte-derived	563 ± 271	71 ± 23
Endothelium-derived	467 ± 139	140 ± 38

Data are reported as mean ± SD. No.: number

**Table 3:** TF^pos^-EVs’ cellular origin in COVID-19 patients.

	TF^pos^-sEVs (No./µL)							TF^pos^-lEVs (No./µL)			
	ACUTE	FU	HS	*p*-Value Acute vs. HS	*p*-Value Acute vs. FU		ACUTE	FU	HS	*p*-Value Acute vs. HS	*p*-Value Acute vs. FU
CD41^pos^	34 ± 7	20 ± 2	17 ± 5	0.002	0.003		159 ± 80	91 ± 59	52 ± 13	0.033	0.042
CD14^pos^	21 ± 6	13 ± 5	15 ± 7	0.149	0.067		34 ± 11	29 ± 14	16 ± 4	0.017	0.371
CD66^pos^	23 ± 5	11 ± 3	11 ± 2	0.001	0.002		78 ± 47	49 ± 24	21 ± 12	0.639	0.112

Data are reported as mean ± SD. No.: number

## Data Availability

The data presented in this study are available from the corresponding author upon request.

## Abbreviations

sEV: small extracellular vesicles; lEVs: large extracellular vesicles; TF: tissue factor; TFPI: tissue factor pathway inhibitor; FXa: factor Xa; COVID-19: Coronavirus disease 2019; HSs: healthy subjects; FU: follow-up; FCM: flow cytometry; PFP: platelet-free plasma.
